# Genomic Profiling of Oral Squamous Cell Carcinoma by Array-Based Comparative Genomic Hybridization

**DOI:** 10.1371/journal.pone.0056165

**Published:** 2013-02-14

**Authors:** Shunichi Yoshioka, Yoshiyuki Tsukamoto, Naoki Hijiya, Chisato Nakada, Tomohisa Uchida, Keiko Matsuura, Ichiro Takeuchi, Masao Seto, Kenji Kawano, Masatsugu Moriyama

**Affiliations:** 1 Department of Molecular Pathology, Faculty of Medicine, Oita University, Oita, Japan; 2 Department of Dentistry and Oral-Maxillo-Facial Surgery, Oita, Japan, Faculty of Medicine, Oita University, Oita, Japan; 3 Department of Computer Science/Scientific and Engineering Simulation, Nagoya Institute of Technology, Nagoya, Japan; 4 Division of Molecular Medicine, Aichi Cancer Center Research Institute, Nagoya, Japan; Deutsches Krebsforschungszentrum, Germany

## Abstract

We designed a study to investigate genetic relationships between primary tumors of oral squamous cell carcinoma (OSCC) and their lymph node metastases, and to identify genomic copy number aberrations (CNAs) related to lymph node metastasis. For this purpose, we collected a total of 42 tumor samples from 25 patients and analyzed their genomic profiles by array-based comparative genomic hybridization. We then compared the genetic profiles of metastatic primary tumors (MPTs) with their paired lymph node metastases (LNMs), and also those of LNMs with non-metastatic primary tumors (NMPTs). Firstly, we found that although there were some distinctive differences in the patterns of genomic profiles between MPTs and their paired LNMs, the paired samples shared similar genomic aberration patterns in each case. Unsupervised hierarchical clustering analysis grouped together 12 of the 15 MPT-LNM pairs. Furthermore, similarity scores between paired samples were significantly higher than those between non-paired samples. These results suggested that MPTs and their paired LNMs are composed predominantly of genetically clonal tumor cells, while minor populations with different CNAs may also exist in metastatic OSCCs. Secondly, to identify CNAs related to lymph node metastasis, we compared CNAs between grouped samples of MPTs and LNMs, but were unable to find any CNAs that were more common in LNMs. Finally, we hypothesized that subpopulations carrying metastasis-related CNAs might be present in both the MPT and LNM. Accordingly, we compared CNAs between NMPTs and LNMs, and found that gains of 7p, 8q and 17q were more common in the latter than in the former, suggesting that these CNAs may be involved in lymph node metastasis of OSCC. In conclusion, our data suggest that in OSCCs showing metastasis, the primary and metastatic tumors share similar genomic profiles, and that cells in the primary tumor may tend to metastasize after acquiring metastasis-associated CNAs.

## Introduction

Oral squamous cell carcinoma (OSCC), which accounts for more than 90% of all oral cancers, is the most common type of head and neck squamous cell carcinoma (HNSCC), and in 2008 was responsible for about 128,000 deaths worldwide [1]. The presence of cervical lymph node metastasis is associated with a 50% decrease in the 5-year survival of patients with OSCC [2], [3]. Therefore, it is important to detect or predict the presence of lymph node metastasis in order to treat OSCC effectively. However, even examinations using imaging techniques such as CT, MRI and ultrasonography are still not reliable for detection of micrometastases because of the high incidence of occult neck disease [4]–[11]. Furthermore, although many parameters of the primary tumors, such as size, thickness and altered gene expression [12]–[18], have been reported to be useful for identifying node-negative patients with a high risk of harboring occult node metastasis [18], the mechanisms by which tumor cells spread from the primary site to local lymph nodes are not well understood [19].

Early studies of colorectal and pancreatic cancers led to a notion that the development and progression of these cancers are associated with accumulation of chromosomal aberrations, a concept referred to as the multistep tumorigenesis model [20]. In fact, it has been reported that the total number of genomic aberrations increases with tumor progression in various tumor types [21]. Meanwhile, recent studies have established extension of the model, designated the clonal evolution model [22]–[25], in which a single clone evolves into several distinct subpopulations through accumulation of diverse genetic abnormalities. The predominant population may be replaced by distinct subpopulations within a single tumor mass through the effects of environmental selection pressure and/or the stage of tumor progression. As a consequence, several genetically heterogeneous subpopulations coexist within a single tumor mass. Despite the emergence of these tumor progression models, understanding of the molecular and cellular mechanisms underlying lymph node metastasis is still limited [26], [27]. Since 1) the break point and patterns of genomic copy number aberrations (CNAs) tend to be reproduced in descendant clones, and 2) genomic aberrations contribute to the malignant behavior of tumor cells by dysregulating the expression of oncogenes or tumor suppressor genes [28], comparison of genomic profiles between a primary tumor and its paired metastasis should provide clues to the process and mechanism of metastasis.

Array-based comparative genomic hybridization (array CGH) provides information about genomic copy number aberrations (CNAs) across the entire genome [29]. Array CGH is generally used to identify oncogenic or tumor-suppressive genes located in regions showing copy number aberration. Moreover, CGH can also be applied to studies of tumor clonality by collecting multiple samples from within a single tumor [30]–[33]. Although several groups have used array CGH to identify regions harboring oncogenic or tumor-suppressive genes in OSCC [34]–[40], the relevance of CNAs in the process of lymph node metastasis has not yet been fully characterized. In OSCC, only one study has compared the genomic profiles of the primary tumor and its associated metastases using array CGH [38]. However, that study included a relatively small number of cases (8 cases) and did not compare the clonality between the genomic profiles of the primary and the metastasis in each case. In HNSCC, a few studies have analyzed the clonal relationship between primary and metastatic tumors [41]–[43]. However, since those studies used conventional metaphase CGH, which has limited resolution, details of genomic regions showing similarities and differences between the two sites were not fully characterized.

Our aim in the present study was to investigate genetic relationships, such as clonality and heterogeneity, between primary tumors of OSCC and their corresponding metastases using high-resolution array CGH, and to identify genomic CNAs related to lymph node metastasis. For these purposes, we collected tumor samples from the metastatic primary tumors (MPTs), their paired cervical lymph node metastases (LNMs), and non-metastatic primary tumors (NMPTs), analyzed their genomic profiles by array CGH, and compared these profiles between MPTs and paired LNMs, and between LNMs and NMPTs.

## Materials and Methods

### Ethics Statement

This study was approved by the ethics committee of Oita University Hospital (Approval No 520 and P-09-03). Informed written consent was obtained from all patients and/or their families.

### Patients, Tissue Samples and Extraction of Genomic DNA

**Figure 1:**
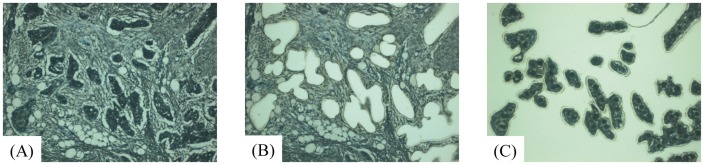
Representative processes of laser-capture microdissection. Tissue sections were stained with toluidine blue for laser-capture microdissection. Tissue sections before and after microdissection are shown in (A) and (B), respectively. Tumor cells harvested on the film are shown in (C).

Twenty-five OSCCs were surgically resected at Oita University Hospital. All patients had sporadic tumors and not multiple primary tumors. Tissue sections were cut from formalin-fixed, paraffin-embedded tissues, and stained with hematoxylin-eosin for histological analysis and with toluidine blue (Wako, Osaka, Japan) for extraction of genomic DNA. Using laser-capture microdissection (Arcturus Engineering, Mountain View, CA, USA) (Figure 1), we collected a total of 42 samples from 25 patients (Table 1 and Table S1), including 15 paired samples of MPTs and their corresponding LNMs, 10 samples of NMPTs, and 2 samples that were separately microdissected from the same tissue section of LNM from case 8 (see Figure S1). All samples included a proportion of tumor cells exceeding 80% of the total. Patients with metastatic OSCCs were selected randomly. However, to reduce any selection bias in terms of tumor thickness (TT), which is known to have a strong association with the risk of lymph node metastasis, we selected non-metastatic OSCCs with a TT of more than 4 mm. As a result, the difference of median TT between MPTs and NMPTs did not reach statistical significance (p = 0.615, Mann-Whitney U test). Genomic DNA was extracted according to the standard proteinase K digestion method, followed by phenol/chloroform extraction. As the source of control DNA, genomic DNA was extracted from tissues of normal renal cortex obtained from 12 patients with ureteral or renal pelvic carcinoma, neither of which exhibited invasion or metastasis to the renal cortex. The same amount of genomic DNA extracted from 12 patients was mixed and used as the control DNA.

**Table 1:** Clinicopathological characteristics of the patients.

Case ID	Age	Sex	Site	Stage	Diff^a^	Meta^b^	Delayed meta^c^	TT^c^ (mm)
Case 1	48	M	tongue	T2N2bM0	well	+	−	11
Case 2	66	M	floor of mouth	T2N2cM0	well	+	−	2
Case 3	71	F	tongue	T2N0M0	well	+	+	6
Case 4	69	F	gingiva	T3N0M0	moderate	+	+	4
Case 5	78	F	tongue	T2N0M0	well	+	+	4
Case 6	89	F	gingiva	T2N0M0	well	+	+	10
Case 7	75	F	tongue	T1N0M0	well	+	+	3
Case 8	62	M	tongue	T2N0M0	well	+	+	9
Case 9	61	M	tongue	T2N0M0	well	+	+	7
Case 10	72	M	buccal mucosa	T2N0M0	well	+	+	6
Case 11	72	F	buccal mucosa	T1N0M0	well	+	+	11
Case 12	74	M	floor of mouth	T1N0M0	moderate	+	+	2
Case 13	68	F	buccal mucosa	T4N0M0	well	+	+	2
Case 14	63	M	tongue	T2N0M0	well	+	+	7
Case 15	57	M	tongue	T1N0M0	well	+	+	7
Case 16	76	F	tongue	T1N0M0	well	−	−	4
Case 17	58	M	tongue	T3N0M0	well	−	−	10
Case 18	44	M	tongue	T2N0M0	well	−	−	11
Case 19	71	M	tongue	T2N0M0	well	−	−	5
Case 20	73	F	tongue	T1N0M0	well	−	−	4
Case 21	83	F	floor of mouth	T2N0M0	moderate	−	−	8
Case 22	74	M	tongue	T2N0M0	well	−	−	10
Case 23	80	M	floor of mouth	T2N0M0	well	−	−	6
Case 24	86	F	tongue	T1N0M0	well	−	−	5
Case 25	75	M	tongue	T1N0M0	well	−	−	4

a Differentiation.

b Metastatic neck lymph node.

c Delayed lymph node metastasis.

d Tumor thickness.

### Array CGH

Array-CGH analysis was performed using 44 k oligonucleotide CGH arrays (Agilent Technologies, Palo Alto, CA, USA). Labeling and hybridization were performed in accordance with the manufacturer’s instructions. Briefly, 1.5–2 µg of tumor DNA and an equal amount of control DNA were digested with AluI and RsaI (Promega, Madison, WI, USA). The digested tumor and control DNAs were labeled with Cy5-dUTP and Cy3-dUTP, respectively, using a Genomic DNA Labeling Kit Plus (Agilent Technologies), purified with Microcon YM-30 filters (Millipore, Billerica, MA, USA), and concentrated to 80.5 µl. Equal amounts of tumor and control DNAs were subsequently pooled and mixed with human Cot-1 DNA, dissolved in hybridization buffer (Agilent Technologies), denatured and hybridized to the CGH array at 65°C for 24 h. Glass slides were washed and then scanned in accordance with the manufacturer’s instructions.

### Data Analysis

Microarray images were analyzed using FEATURE EXTRACTION v.9.5.3.1 (Agilent Technologies) with linear normalization (protocol CGH-v4_95_Feb07), and the resulting data were imported into DNA Analytics v.4.0.8.1 (Agilent Technologies). After normalization of the raw data, the log2ratio of Cy5 (tumor) to Cy3 (control) was calculated. Aberrant regions were determined using the ADM-2 algorithm at a threshold of 7.0. To detect gains and losses, we set the values of parameters for the aberration filters as: minimum number of probes in region 2, minimum absolute average log2ratio for region 0.163, maximum number of aberrant regions 10,000, and percentage penetrance per feature 0. We set the value of the minimum absolute average log2 ratio at 0.163 to detect regions showing a change in the averaged copy number of more than 1.12-fold (log2(1.12) = 0.163). We selected this setting to derive values at least as conservative as the default ones. This was confirmed by a set of reference-versus-reference CGH analyses using the ADM-2 algorithm employing the same aberration filters, in which no aberrant regions were detected (data not shown). This indicated that the estimated false positivity rate was nearly zero, and that our parameter setting was sufficiently conservative. Data generated by probes mapped to the X and Y choromosomes were eliminated. All CNAs detected in each sample and the qualities of array data are summarized in Tables S2 and S3, respectively. All the data are MIAME-compliant, and have been deposited in the GEO database (http://www.ncbi.nlm.nih.gov/geo/, accession number GSE36942). Similarities of genomic profiles between two samples were expressed as concordance rates for probes with which aberrantly gained or lost signals were detected by array CGH. Rates of concordance between paired or non-paired MPTs and LNMs were calculated. In unsupervised hierarchical clustering, we used complete shrinkage as the cluster merge option and (uncentered) correlation as the similarity metric, which were the default settings of the Gene cluster 3.0 software.

### Statistical Analysis

Paired *t* test and Fisher’s exact test were used. Differences at *P*<0.05 were considered to be statistically significant.

## Results

### Close Similarity with a Minor Degree of Heterogeneity between Genomic Profiles of MPTs and Paired LNMs in Metastatic OSCCs

**Figure 2:**
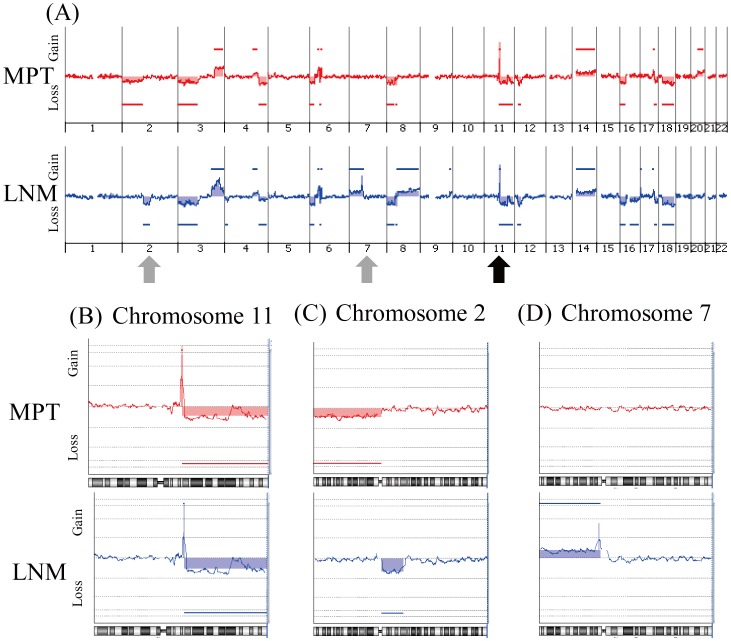
Representative genomic profile of a MPT and the corresponding paired LNM of a metastatic OSCC. Whole genomic profiles of the paired samples of MPT (above) and LNM (below) from case 8 are shown in (A). Detailed genomic profiles of Chr11 indicated by a black arrow in (A), and Chr2 and Chr7 indicated by gray arrows in (A) are shown in (B), (C) and (D), respectively. Horizontal lines above the center represent regions of gain, and those below the center represent regions of loss. Both MPT and LNM show similar genomic patterns in chromosome 11q (B). However, loss of 2p is detected only in the MPT (C), and loss of 2q and gain of 7p are detected only in the LNM (C and D).

To investigate the genetic relationship between MPT and the paired LNM in each case, we analyzed the genomic profiles of 15 MPT-LNM pairs using array CGH. One representative case (Case 8) is shown in Figure 2. The MPT and paired LNM of this case shared a similar profile pattern across the entire genome (Figure 2A), especially in chromosome 11q (Figure 2B), suggesting that tumor cells in the MPT and paired LNM of this case were clonally related. In the same case, however, there were also distinct genomic aberrations in chromosomes 2p, 2q, 7p, 8q, 16q and 20q (Figure 2A). Loss of 2p was observed only in the MPT, and not in the LNM, and loss of 2q and gain of 7p were observed only in the LNM, and not in the MPT (Figure 2C and D), suggesting that the MPT and paired LNM in this case were composed of genetically distinct subpopulations. To show the geographical distribution of subclones in the same tissue section, we performed separate dissection on tissue sections of LNM from case 8. As shown in Figure S1A, B and C, we collected tumor cells from two sites on the LNM tissue sections and analyzed genomic aberrations by array CGH. Although we were unable to find any clear difference between the patterns of genomic aberration of the two samples (Figure S1D), we found differences in the log2 ratio of aberrations. As shown in Figure S1E, the intensity of 11q13 amplification was slightly higher in LNM2 than in LNM1 (averaged log2 ratios are 3.2 and 2.1, respectively, indicated by shaded areas), while that of 7p12 amplification was clearly higher in LNM1 than in LNM2 (averaged log2 ratios are 2.4 and 1.2, respectively, Figure S1F). These results suggest that these two sites may be composed of clonally related but genetically distinct subclones. Additionally, the aberration pattern of chromosome 3q of LNM2 was more similar to that of PMT than to that of LNM1 (Figure S1G), suggesting that tumor cells in LNM2 may be more closely related to those in PMT.

**Figure 3:**
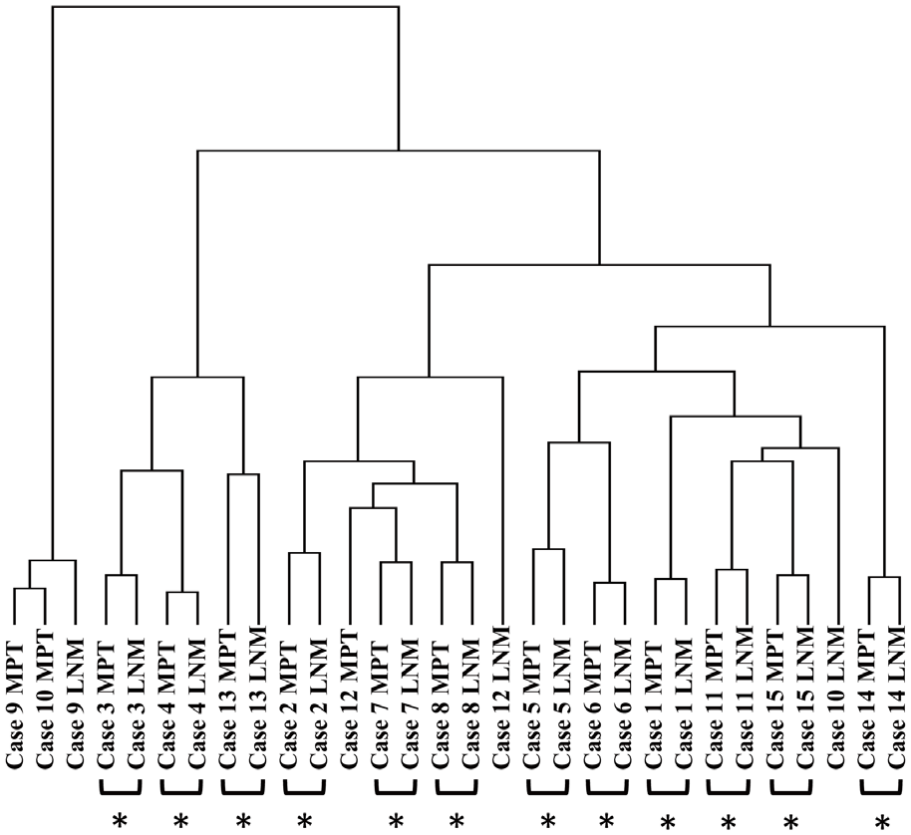
Unsupervised hierarchical clustering of genomic CNAs in MPT and LNM tumor samples from 15 patients. Unsupervised hierarchical clustering for the 30 tumor samples from 15 patients was performed based on the genomic CNAs. Twelve of 15 the MPT-LNM pairs were clustered together (indicated by asterisks).

**Figure 4:**
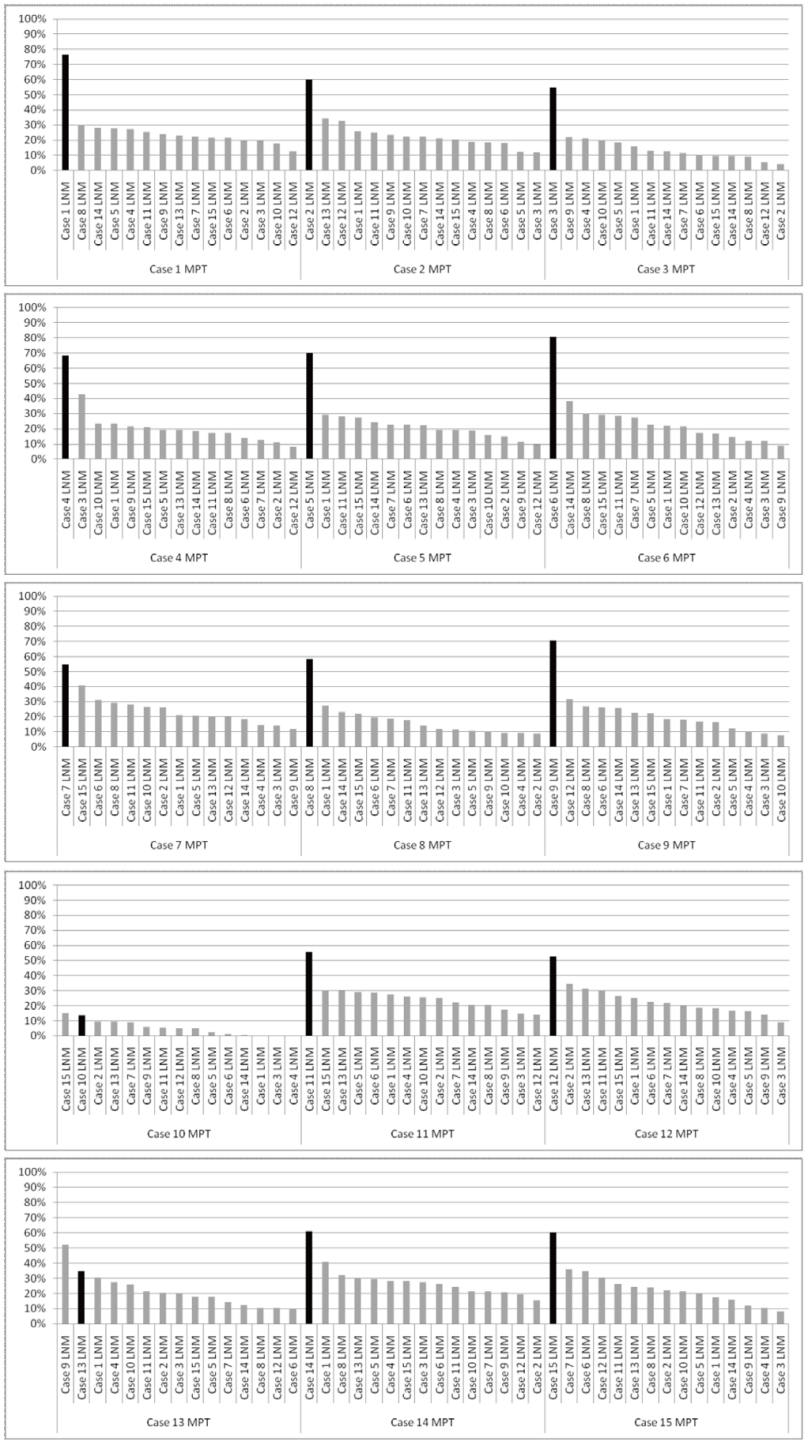
Concordance rates of genomic profiles between MPTs and LNMs. Concordance rates of genomic profiles between MPTs and the paired LNMs (intra-case comparison), and MPTs and LNMs in the other 14 cases (inter-case comparison) were calculated as described in Materials and Methods. The concordance rates for intra-case and inter-case comparisons are shown by black- and gray-colored bars, respectively.

We also compared the genomic profiles of the other 14 paired MPT and LNM samples individually. Although some distinctive differences in genomic profiles were observed between MPTs and paired LNMs, the paired samples in all cases showed similar patterns of CNAs across the entire genome (Figures S2, S3 and S4). Furthermore, unsupervised hierarchical clustering based on array CGH data from MPTs and LNMs grouped together 12 of the 15 pairs (Figure 3), indicating that the genomic profile of the LNM was most similar to that of the paired MPT for the 12 clustered pairs. In addition, we analyzed the significance of similarities between MPTs and paired or non-paired LNMs by calculating the concordance rates (See Materials and Methods). As shown in Figure 4, the concordance rate was highest between the MPT and the paired LNM in 13 of the 15 cases. The median of the concordance rate between paired samples was significantly higher than that between non-paired samples (Mann Whitney U test, p<0.01). With a few notable exceptions, such as case 10, in which CNAs of paired MPT and LNM differed the most in clustering analysis (Figure 3) and did not show highest similarity between the paired samples (Figure 4), our results suggested that MPTs and paired LNMs contain predominantly clonal tumor cells, while minor subpopulations with different CNAs may also exist in metastatic OSCCs.

**Figure 5:**
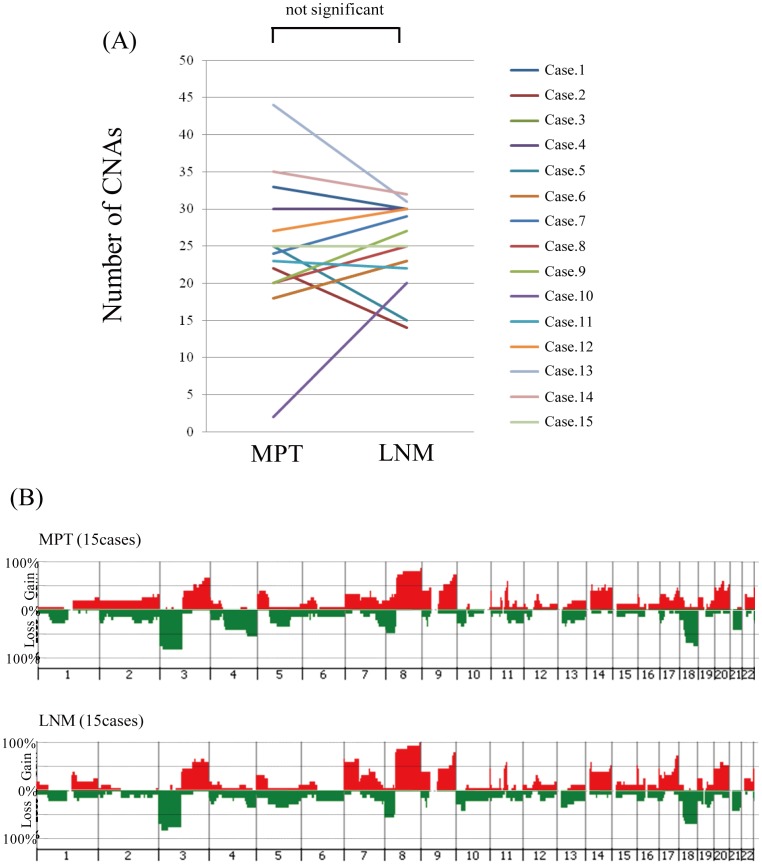
Comparison of CNAs between MPTs and the corresponding LNMs. (A) Comparison of the number of CNAs in the MPT and corresponding LNM in each case. (B) Genome-wide frequencies of CNAs in the MPTs and corresponding paired LNMs in the 15 cases. Horizontal lines: oligonucleotide probes are shown in order from chromosomes 1 to 22. Within each chromosome, clones are shown in order from the p telomere to the q telomere. Vertical lines: frequency (%) of gains (positive axis) and losses (negative axis) are shown for each probe.

Next, to determine whether acquisition of CNAs is required for spread of tumor cells from the primary site to regional lymph nodes, we compared the number of CNAs between MPTs and paired LNMs. Seven of the 15 cases showed an increased number of CNAs in the LNM, 6 cases showed a decrease, and the remaining 2 cases showed no change (Figure 5A). As a result, there were no significant differences in the number of CNAs between MPTs and paired LNMs (Figure 5A, p = 0.742). Furthermore, to identify CNAs related to lymph node metastasis, we compared the frequencies of CNAs between grouped samples of MPTs and LNMs (Figure 5B), but were unable to find any CNAs that were significantly more common in LNMs (Table S4). Thus, our data suggested that additional CNAs are not necessarily required in order for tumor cells to spread from the primary site to regional lymph nodes in OSCC.

### Identification of Metastasis-associated CNAs in OSCCs

**Figure 6:**
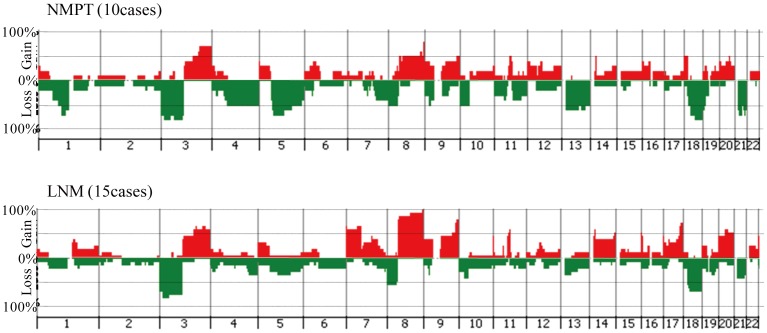
Genome-wide frequencies of CNAs in 10 NMPTs and 15 LNMs. Horizontal lines: oligonucleotide probes are shown in order from chromosomes 1 to 22. Within each chromosome, clones are shown in order from the p telomere to the q telomere. Vertical lines: frequency (%) of gains (positive axis) and losses (negative axis) are shown for each probe.

Since no statistically significant differences were detected in the frequencies of CNAs between MPTs and paired LNMs (Figure 5B), we hypothesized that subpopulations carrying metastasis-related CNAs might be present in MPTs as well as LNMs. Furthermore, it was possible that MPTs might also contain non-metastatic subpopulations. For example, in case 8, loss of 2p was detected only in the MPT, and not in the LNM (Figure 2C). Therefore, to detect specific CNAs involved in cervical lymph node metastasis, we compared the genomic profiles of NMPTs (Figures S5 and S6) and LNMs of metastatic OSCC. As shown in Figure 6, gains at 3q, 9q,11q13, 14q, 16p, 18p and 20q, and losses at 3p, 4q, 8p, 9p, 10p, 13q, 18q and 21q were detected at a frequency of more than 50% in both NMPTs and LNMs (Table S5), suggesting that these CNAs may be involved in the development of OSCC. On the other hand, gains at 7p, 8q and 17q were differentially detected in LNMs (p<0.05, Figure 6 and Table 2), suggesting that these CNAs may be involved in the lymph node metastasis of OSCC. Interestingly, losses at 1p, 5q, 9p and 19p were detected more frequently in NMPTs than LNMs (p<0.05, Figure 6 and Table2), suggesting that these CNAs may be related to a non-metastatic phenotype of OSCC cells.

**Table 2:** Genomic CNAs with potential candidate genes found to differ significantly between LNMs and NMPTs.

	Chromosomal region (bp)		LNM	NMPT	Fisheŕs exact test	
Chromosomal band	start	stop	n = 15	n = 10	p-value	Candidate genes
***(Gains)***						
**7p**						
7p22.3	797378	1163891	8	1	0.04	CYP2W1
7p22.2	2167170	3093625	10	2	0.041	MAD1L1,NUDT1
7p22.1–p14.1	6006721	42940867	9	1	0.018	RAC1, TWIST1, EIF2AK1, ETV1, AGR2, NPY, HOXA1
7p13	43765914	45112454	10	2	0.041	CCM2
7p13–p12.3	45190313	49100735	10	1	0.012	IGFBP3
7p12.3–p12.1	49282714	51855880	10	2	0.041	DDC
7p12.1–p11.2	52035557	53934357	10	1	0.012	
7p11.2	54293570	56115221	10	2	0.041	EGFR, SEC61G
**8q**						
8q11.22–q12.1	50929121	59427474	13	4	0.028	PLAG1, RP1
8q22.1–q24.21	94619246	129216964	14	5	0.023	MYC, TNFRSF11B, MTDH, CCNE2
8q24.22–q24.3	134010490	142030549	14	5	0.023	PTK2, NDRG1
**17q**						
17q24.3	67021962	67198033	8	1	0.04	
17q24.3–q25.1	67580412	68713194	9	1	0.018	SSTR2, SOX9
17q25.1	69982273	70367509	10	2	0.041	
17q25.1–q25.3	70376047	78154478	11	2	0.015	BIRC5, GRB2, ITGB4, SPHK1, TK1, TIMP2, FASN
17q25.3	78189789	78218020	10	1	0.012	
17q25.3	78238617	78478382	9	1	0.018	
17q25.3	78491903	78586290	8	1	0.04	
***(Losses)***						
1p22.1–p13.3	93771144	109201241	3	7	0.034	
5q11.2–q14.2	58331366	82590164	4	7	0.049	
9p13.3–p13.1	35746179	38612248	0	4	0.017	
19p13.3	544916	2001171	1	5	0.023	
19p13.3	2280486	2429166	2	6	0.028	

## Discussion

In this study, the highest similarity of genomic profiles was observed between MPTs and paired LNMs in 13 out of 15 cases of metastatic OSCC (Figure 4). Furthermore, unsupervised hierarchical clustering grouped 12 of the 15 MPT-LNM pairs (Figure 3), suggesting that the MPT and paired LNM of each metastatic OSCC may share a similar genomic profile pattern. In fact, we were unable to find any significant difference between the averaged frequencies of CNAs in MPTs and those in LNMs (Figure 5B). Meanwhile, we found that all of the cases of metastatic OSCC showed some distinctive genomic CNA patterns between the MPT and paired LNM (Figure S2, S3 and S4). Of note, CNAs that were detected specifically in the MPT but undetected in the LNM were found in 11 of the 15 metastatic OSCCs, suggesting that not all of the CNAs detected in the MPT are inherited by the tumor cells in the paired LNM. For example, in case 8, loss of 2p was observed only in the MPT, and not in the paired LNM (Figure 2C). These results imply the co-existence of genetically distinct subpopulations within a single tumor mass of metastatic OSCC. Thus, the findings presented here suggest that although we cannot exclude the possibility that small genetically heterogeneous subpopulations may be admixed in metastatic OSCC, the MPT and paired LNM are composed predominantly of genetically clonal tumor cells.

**Figure 7:**
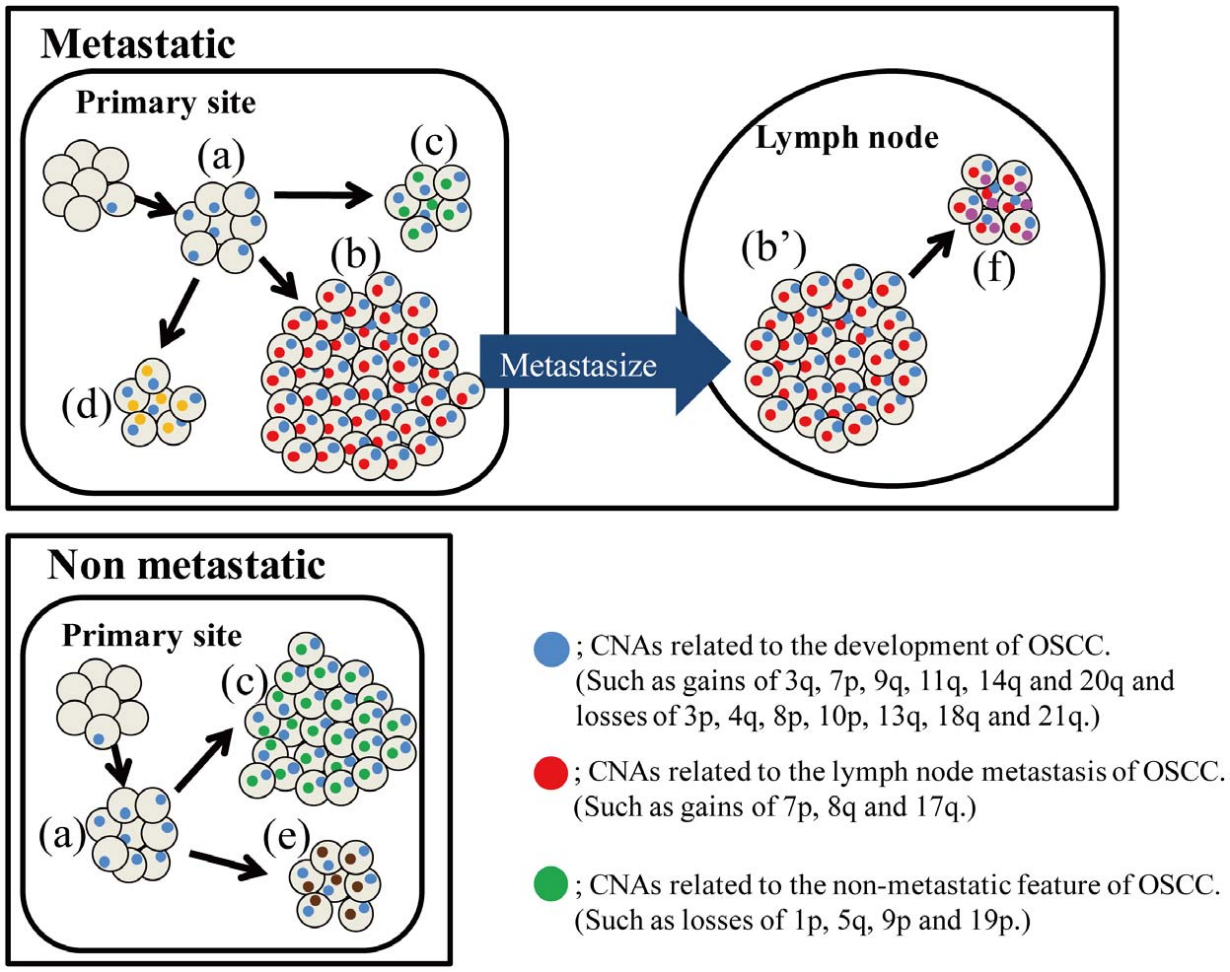
Hypothetical model for the development of metastatic OSCC. Clear gray circles indicate cells. Small colored circles indicate genomic aberrations. OSCC arises from a single cell with one (or a few) genomic aberrations. The single clone then proliferates more effectively than its neighbors (a). During the process of proliferation, some tumor cells acquire additional mutations at random. Subsequently, each of the genetically distinct subclones forms a unique subpopulation in the primary tumor (b, c, d and e). Among these subpopulations, only those with the capacity for metastasis can spread to cervical lymph nodes (b and b’). After metastasis, one (or a few) subpopulation may again develop further genetically distinct subpopulations through clonal evolution (f). In the advanced stage of progression, the primary tumor and metastases become predominantly populated by single clones with the capacity for metastasis (b and b’).

Based on these findings, we propose a hypothetical model for the development of metastatic OSCC (Figure 7). In this model, the MPT is composed of genetically heterogeneous subpopulations. Among them, subpopulations having metastasis-related CNAs, indicated as small red circles in Figure 7, might metastasize to lymph nodes and form a large part of the subsequent LNM. Indeed, cases with metastasis-related CNAs in MPT, including gains at 7p, 8q and 17q, always harbored the same CNA in the paired LNM (data not shown). In the LNM, one (or a few) subpopulations may again develop further genetically distinct subpopulations through clonal evolution (Figure 7f). To confirm this hypothesis, further studies with larger samples will be required. Interestingly, our model is compatible with the “punctuated clonal evolution” model established by Navin et al. [44]. They aimed to reveal the tumor population structure and evolution in two cases of breast cancer by single-cell sequencing and found that some genetically distinct subpopulation with few persistent intermediate subclones reside within a single primary tumor. Their data suggest that tumor cells whose rate of population growth markedly exceeds its rate of genomic evolution can form a subpopulation in a single tumor. Although no previous study has investigated the clonal evolution of OSCC by deep sequencing, including single-cell sequencing, our hypothesis regarding the progression of OSCC might be verified by these sequencing methods in the future.

In this study, we detected gains at 7p, 8q and 17q more frequently in LNMs than in NMPTs, suggesting that these CNAs are associated with lymph node metastasis. Gain at 7p has already been reported to be involved in lymph node metastasis of OSCC [45], [46]. Although gain of 8q is frequently detected in OSCCs [37], [47], it remains to be determined whether this CNA is actually related to lymph node metastasis of OSCC. In this study, 17q gain was identified as a new candidate CNA related to lymph node metastasis of OSCC. Little is known about the relationship between 17q gain and lymph node metastasis of OSCC. In these regions, many cancer-related genes were located, as summarized in Table 2. Among them, EGFR(7p11.2) [48], TWIST1(7p21.2) [49], RAC1(7p22) [50], MTDH(8q22.1) [51], CCNE2(8q22.1) [52], TNFRSF11B(8q24) [53] and GRB2(17q25) [54] might be good candidates, as they are reportedly associated with invasiveness and metastasis of tumor cells. Further analysis, including gene expression and functional analysis, will be required to clarify this issue. Several previous studies have identified lymph node metastasis-associated CNAs in OSCC [38], [45], [46], [55]: Chen et al. identified gain at 7p, 11q13 and 20q, Liu et al. identified 11q13 gain, Pathare et al. identified 7p gain and 8p loss, and Sugahara et al. identified 11q13 gain. These differences may be attributable to differences in sample sizes, comparison methods, and platforms of CGH employed. Among these CNAs, gains at 7p and 11q13 have been identified as lymph node metastasis-related CNAs in multiple studies (>3) including ours, suggesting that these CNAs may play important roles in the metastasis of OSCC. In the present study, gain at 7p was also identified as a lymph node metastasis-related CNA, but gain at 11q13 was not. At the present time, we cannot fully account for this difference between our results and those of others, but one plausible explanation could be that other groups selected non-metastatic OSCC samples randomly, whereas we selected tumors with a TT of more than 4 mm. Whether these differences in findings are due simply to the different criteria employed for the selection of NMPT samples remains to be clarified.

In this study, a high frequency (50–86%) of metastatic OSCCs with gains at 7p, 8q or 17q in LNMs also harbored the same CNA in the paired MPTs. That is, 10 out of 15 cases showed gain at 7p in the LNM and 5 of those 10 cases also had this CNA in the paired MPT (50%). Similarly, 14 out of the 15 cases showed gain at 8q in the LNM and 12 of the 14 cases also had this CNA in the paired MPT (86%). Eleven of the 15 cases showed 17q gain in the LNM, and 7 of those 11 cases also had this CNA in the paired MPT (64%). These results suggest that patients may have a high risk of lymph node metastasis when gains at these CNAs are detected in the primary tumor, even if the patients are diagnosed as having node-negative OSCC. This suggestion is important in the context of diagnosis and treatment of OSCC, because the majority of current therapeutic strategies are decided on the basis of the primary tumor. More extensive development of our data will be required to identify predictive markers of occult LNM in OSCC.

In this study, we found that gains at 3q, 9q,11q13, 14q, 16p, 18p and 20q, and losses at 3p, 4q, 8p, 9p, 10p, 13q, 18q and 21q were detected at a high frequency in both NMPTs and LNMs of OSCC (Figure 6). To determine whether this tendency is generally observed in OSCC analysis, we compared the frequencies of CNAs in this study with those of publicly deposited CGH arrays (Table S6) [56]. As shown in Figure S7, although most CNAs were detected at a relatively lower frequency than in our analysis, the pattern of the histogram, in which gains at 3q, 11q13 and 20q, and losses at 3p, 8p and 18q were highly detected in 228 OSCC, was similar to that obtained with our data, suggesting that these CNAs may be important for the development of OSCC. On the other hand, some inconsistencies were also observed in the frequency of 8q and 17q gain. We are unable to explain this discrepancy at the present time because of lack of information about lymph node status in the deposited data.

In conclusion, our results suggest that primary tumors of OSCC and their corresponding LNMs share very similar patterns of genomic CNAs, and that cells in the primary tumor tend to become metastatic after acquiring metastasis-associated CNAs, such as gains at 7p, 8q and 17q, during the process of clonal evolution. Further studies will be required to identify the responsible genes located on these CNAs and to clarify the mechanisms underlying the process of lymph node metastasis. This approach may make it possible to predict and treat LNMs by determining whether metastasis-associated CNAs are detectable in biopsy samples from patients with OSCC.

## Supporting Information

Figure S1 **Representative genomic profiles of the two distinct areas of LNM tissue from case 8.** HE staining of LNM tissue from case 8 is shown in low- (A) and high- (B and C) power views. Tumor cells in the area of LNM1 (B) and LNM2 (C) were collected separately using laser-capture microdissection and then subjected to array CGH analysis. Whole-genomic profiles of tumor cells collected from LNM1 and LNM2 are shown in (D). Detailed genomic profiles of 11q13 indicated by a black arrow in (D), 7p11 and Chr3 indicated by gray arrows in (D) are shown in (E), (F) and (G), respectively. Horizontal lines above the center represent regions of gain, and those below the center represent regions of loss. The log2 ratios of amplifications at 11q13 and 7p11 are shown in (E) and (F).
(TIF)

Figure S2 **Whole genomic profiles of paired MPTs (above) and LNMs (below) from 5 cases.** Horizontal lines above the center represent regions of gain, and those below the center represent regions of loss. A black arrow indicates a common pattern. A gray arrow indicates a different pattern.
(TIF)

Figure S3 **Whole genomic profiles of paired MPTs (above) and LNMs (below) from 5 cases.** Horizontal lines above the center represent regions of gain, and those below the center represent regions of loss. A black arrow indicates a common pattern. A gray arrow indicates a different pattern.
(TIF)

Figure S4 **Whole genomic profiles of paired MPTs (above) and LNMs (below) from 4 cases.** Horizontal lines above the center represent regions of gain, and those below the center represent regions of loss. A black arrow indicates a common pattern. A gray arrow indicates a different pattern.
(TIF)

Figure S5 **Whole genomic profiles of the NMPTs from 5 cases.** Horizontal lines above the center represent regions of gain, and those below the center represent regions of loss.
(TIF)

Figure S6 **Whole genomic profiles of the NMPTs from 5 cases.** Horizontal lines above the center represent regions of gain, and those below the center represent regions of loss.
(TIF)

Figure S7 **Genome-wide frequencies of CNAs in 228 OSCCs from Arraymap database website.** Frequencies (%) of gains (positive axis) and losses (negative axis) in 228 OSCCs listed in the Arraymap database website, www.arraymap.org, are shown.
(TIF)

Table S1 **Sample Information.**
(XLSX)

Table S2 **All CNAs detected in each sample.**
(XLSX)

Table S3 **Quality control metrics of all array samples.**
(XLSX)

Table S4 **Comparison of CNAs between MPT and LNM.**
(XLSX)

Table S5 **Comparison of CNAs between NMPT and LNM.**
(XLSX)

Table S6 **Sample IDs and summarized aberrations of publicly deposited data from Arraymap.**
(XLSX)
