# Supplementary figures and images for: Genomic Profiling of Oral Squamous Cell Carcinoma by Array-Based Comparative Genomic Hybridization
Source: PLoS One. 2013 Feb 14;8(2):e56165. doi: 10.1371/journal.pone.0056165 (PMC3573022; doi:10.1371/journal.pone.0056165)

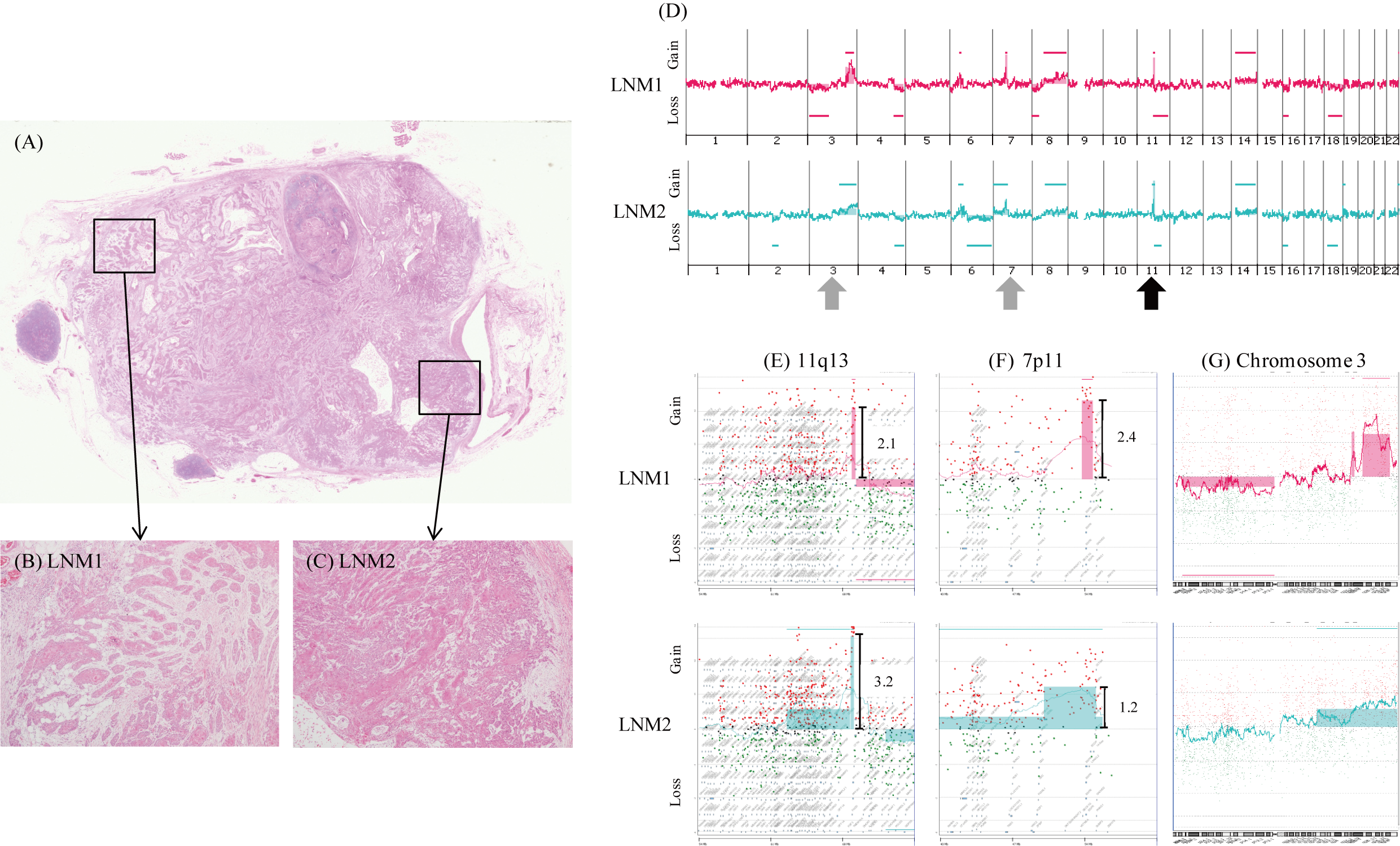

Supplement: Figure S1 — Representative genomic profiles of the two distinct areas of LNM tissue from case 8. HE staining of LNM tissue from case 8 is shown in low- (A) and high- (B and C) power views. Tumor cells in the area of LNM1 (B) and LNM2 (C) were collected separately using laser-capture microdissection and then subjected to array CGH analysis. Whole-genomic profiles of tumor cells collected from LNM1 and LNM2 are shown in (D). Detailed genomic profiles of 11q13 indicated by a black arrow in (D), 7p11 and Chr3 indicated by gray arrows in (D) are shown in (E), (F) and (G), respectively. Horizontal lines above the center represent regions of gain, and those below the center represent regions of loss. The log2 ratios of amplifications at 11q13 and 7p11 are shown in (E) and (F). (TIF) [file pone.0056165.s001.tif]

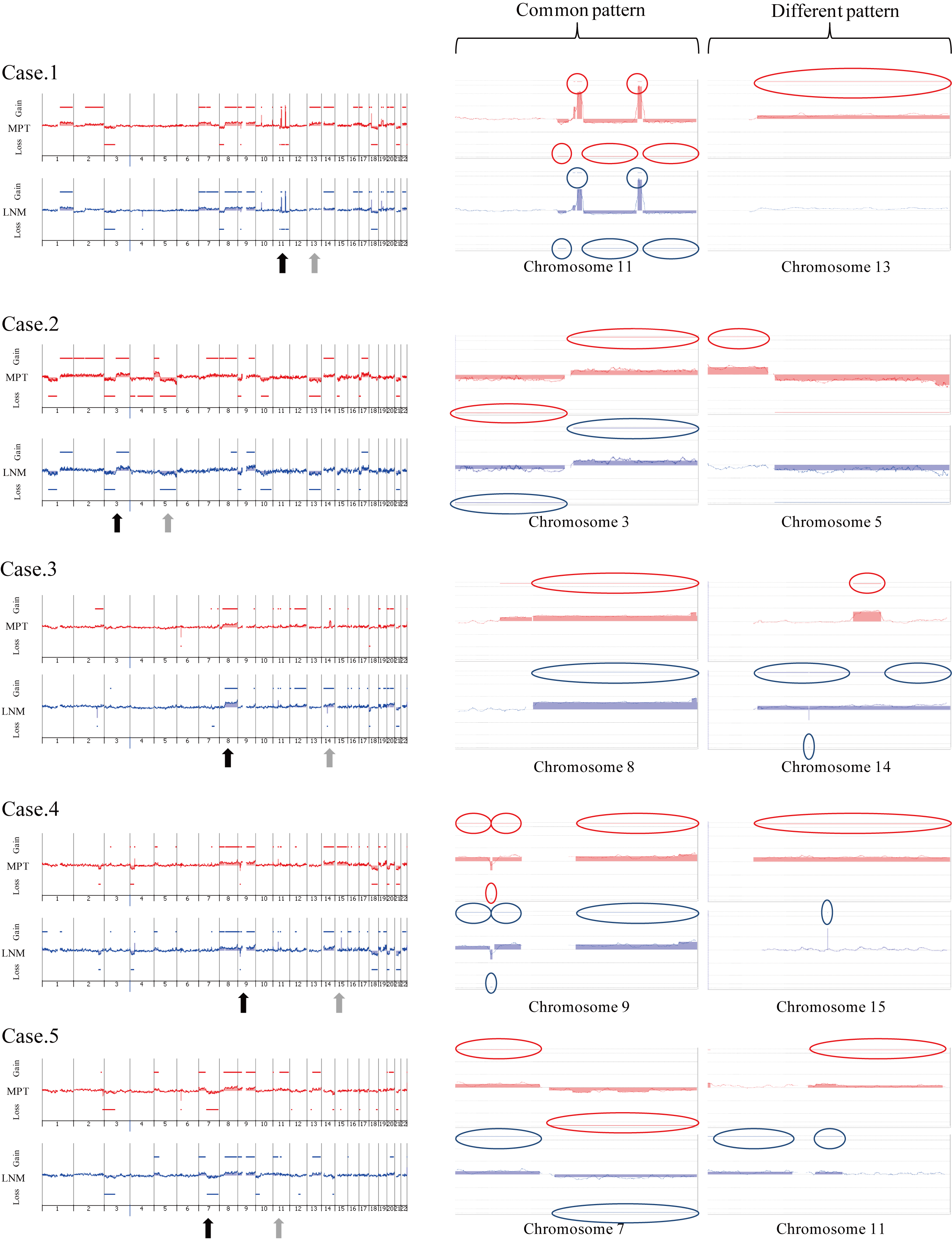

Supplement: Figure S2 — Whole genomic profiles of paired MPTs (above) and LNMs (below) from 5 cases. Horizontal lines above the center represent regions of gain, and those below the center represent regions of loss. A black arrow indicates a common pattern. A gray arrow indicates a different pattern. (TIF) [file pone.0056165.s002.tif]

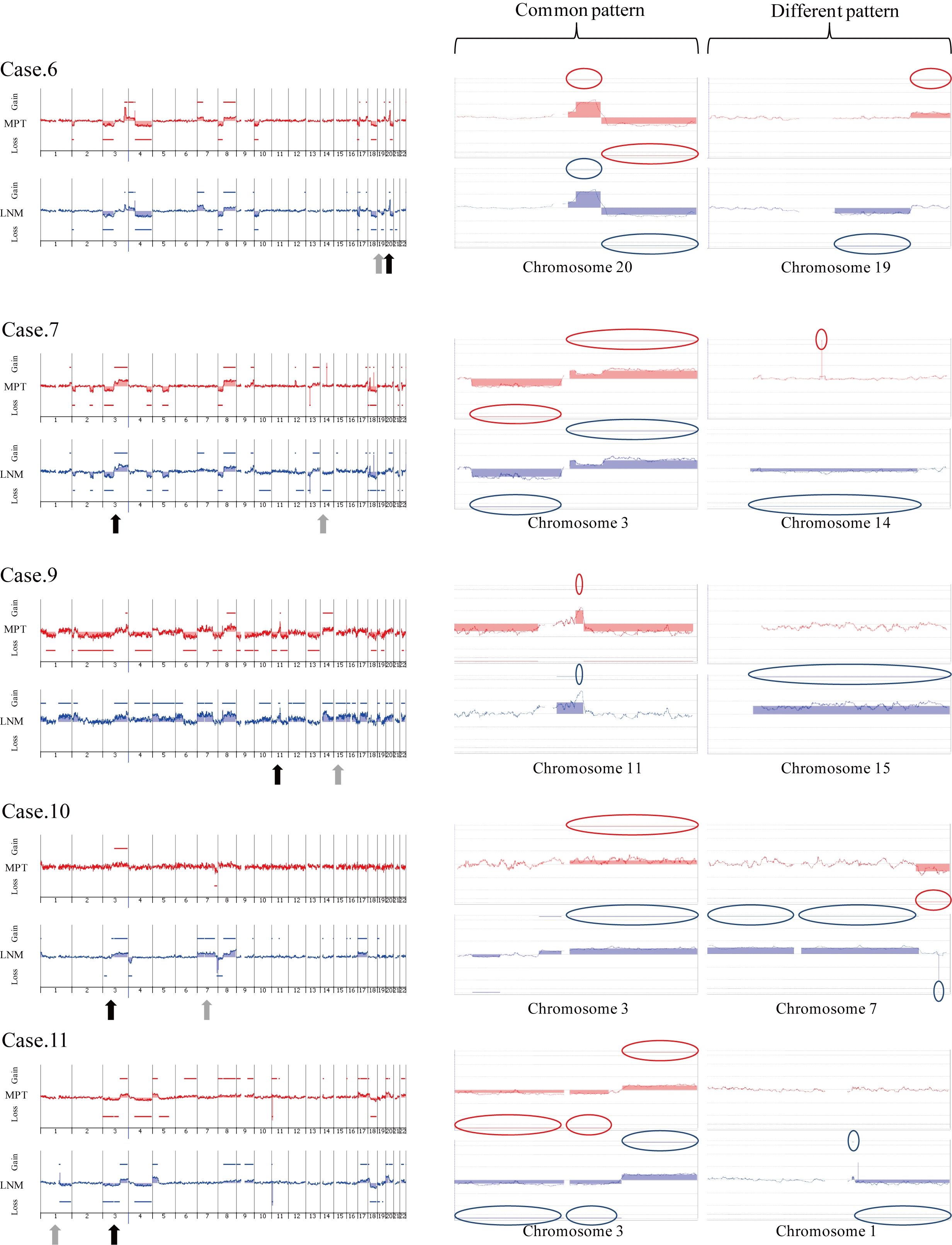

Supplement: Figure S3 — Whole genomic profiles of paired MPTs (above) and LNMs (below) from 5 cases. Horizontal lines above the center represent regions of gain, and those below the center represent regions of loss. A black arrow indicates a common pattern. A gray arrow indicates a different pattern. (TIF) [file pone.0056165.s003.tif]

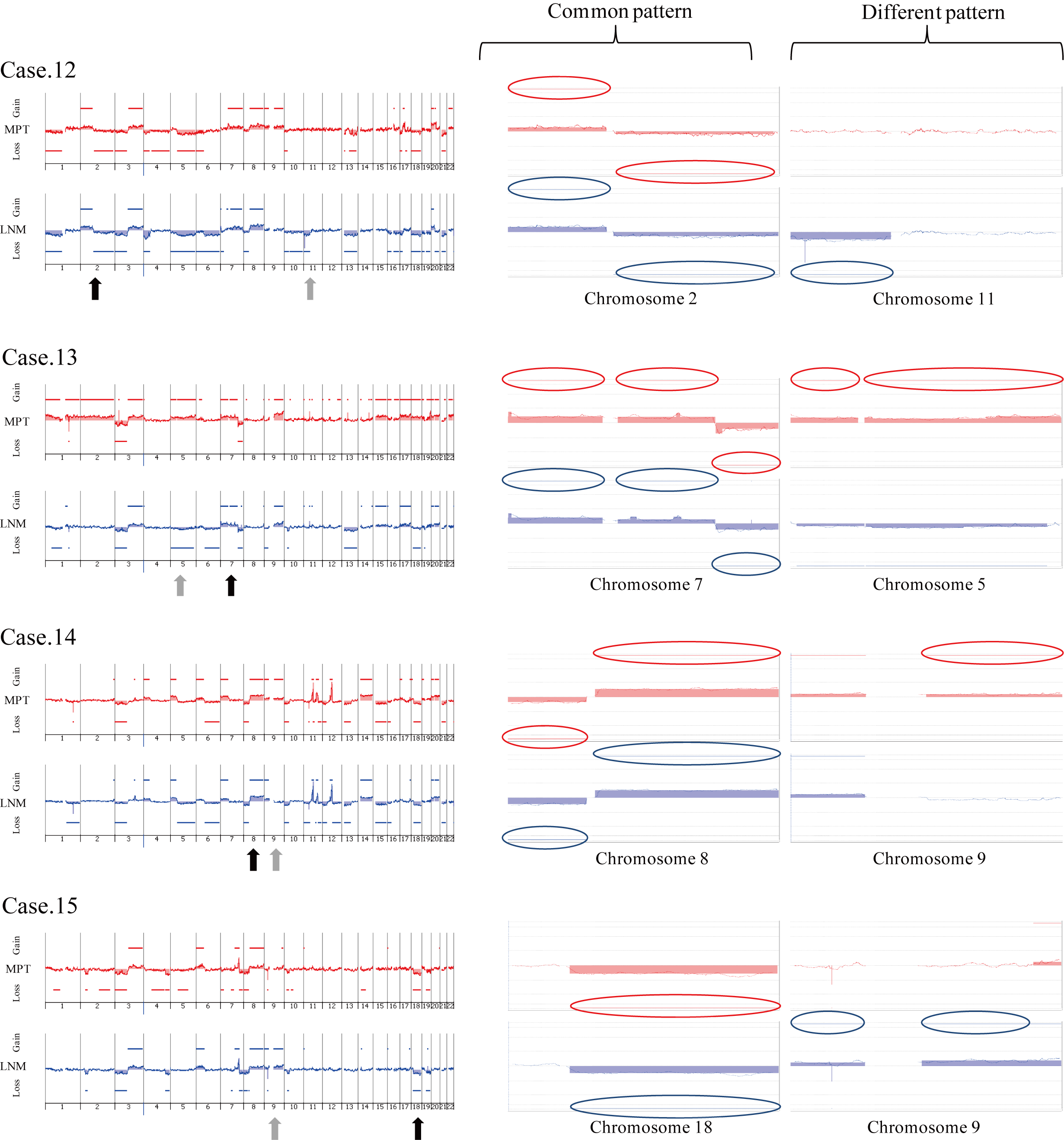

Supplement: Figure S4 — Whole genomic profiles of paired MPTs (above) and LNMs (below) from 4 cases. Horizontal lines above the center represent regions of gain, and those below the center represent regions of loss. A black arrow indicates a common pattern. A gray arrow indicates a different pattern. (TIF) [file pone.0056165.s004.tif]

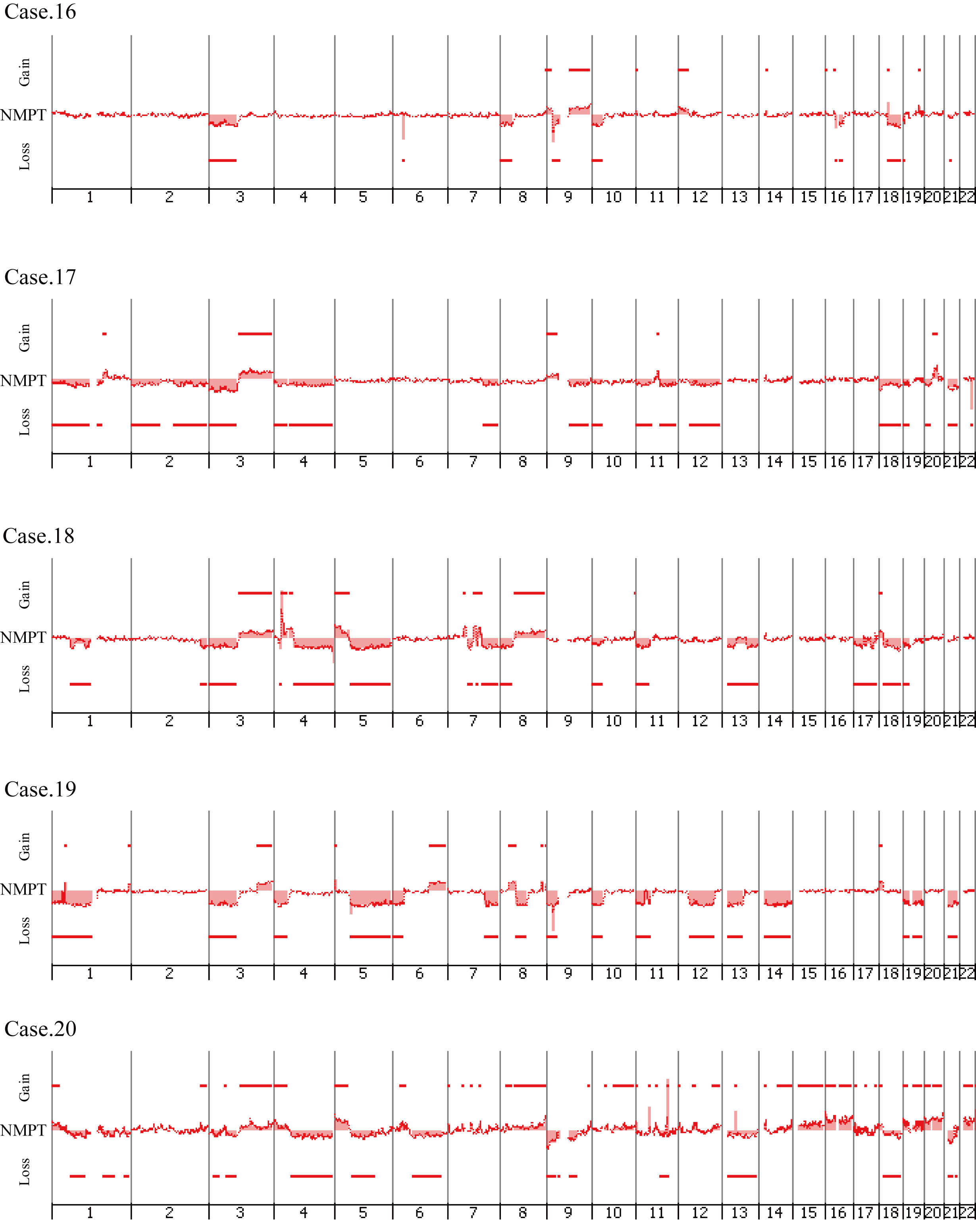

Supplement: Figure S5 — Whole genomic profiles of the NMPTs from 5 cases. Horizontal lines above the center represent regions of gain, and those below the center represent regions of loss. (TIF) [file pone.0056165.s005.tif]

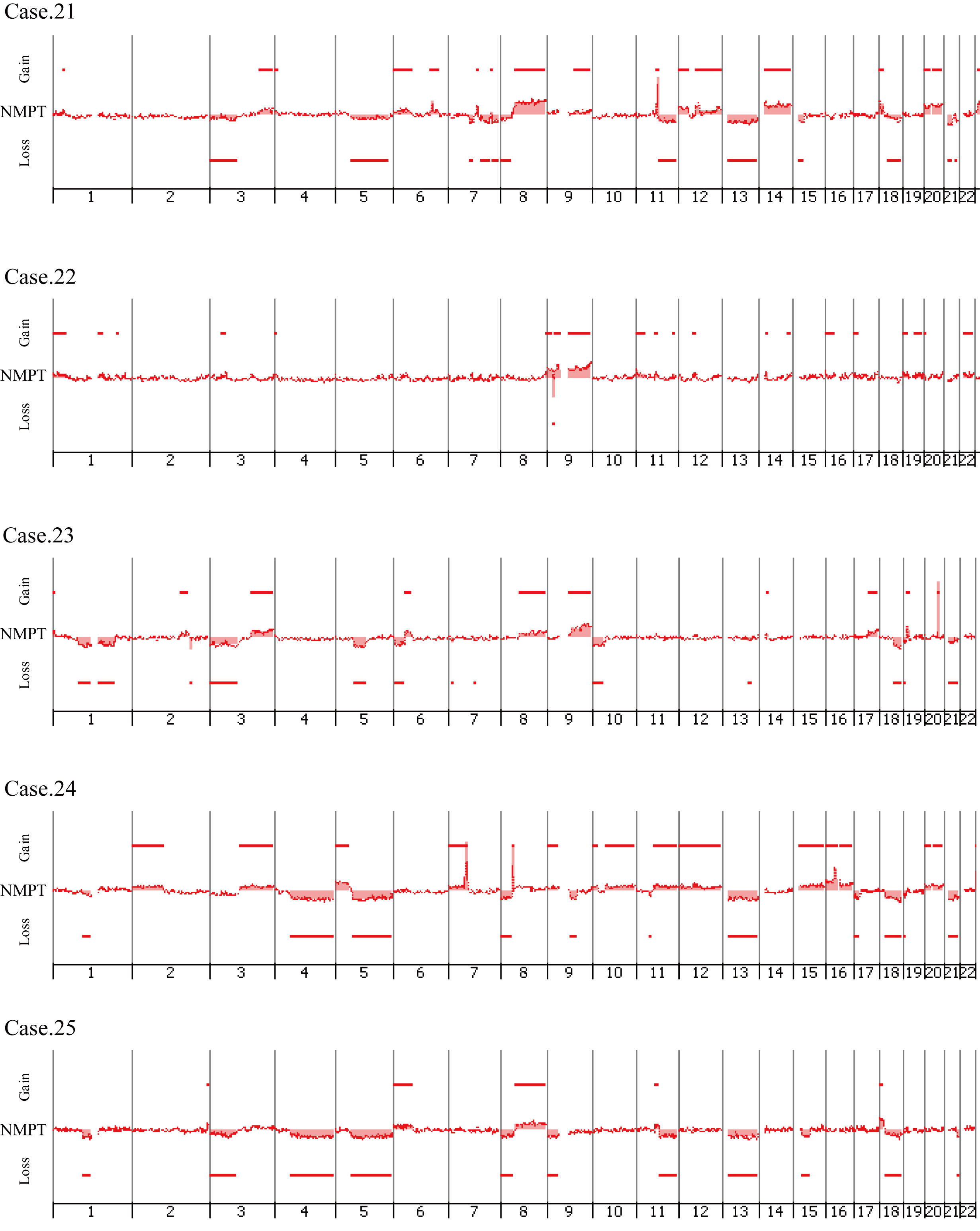

Supplement: Figure S6 — Whole genomic profiles of the NMPTs from 5 cases. Horizontal lines above the center represent regions of gain, and those below the center represent regions of loss. (TIF) [file pone.0056165.s006.tif]

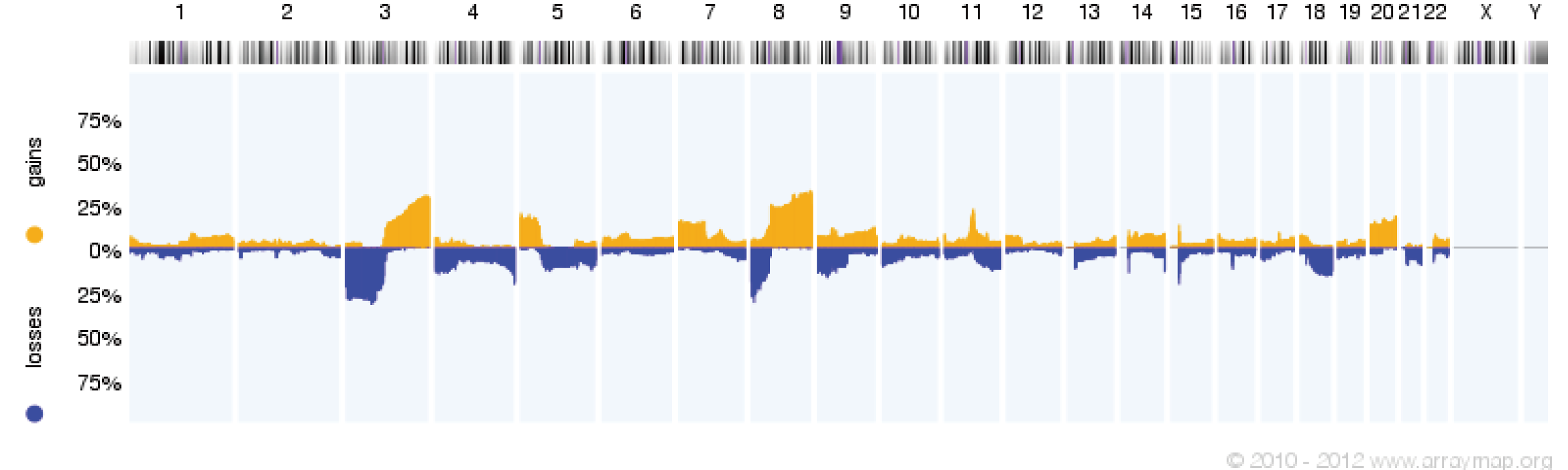

Supplement: Figure S7 — Genome-wide frequencies of CNAs in 228 OSCCs from Arraymap database website. Frequencies (%) of gains (positive axis) and losses (negative axis) in 228 OSCCs listed in the Arraymap database website, www.arraymap.org, are shown. (TIF) [file pone.0056165.s007.tif]
